# Epinephrine auto-injector prescriptions to food-allergic patients in primary care in The Netherlands

**DOI:** 10.1186/s13223-015-0094-9

**Published:** 2015-10-15

**Authors:** Jacquelien Saleh-Langenberg, A. E. J. Dubois, F. Groenhof, J. W. H. Kocks, T. van der Molen, B. M. J. Flokstra-de Blok

**Affiliations:** Department of Pediatric Pulmonology and Pediatric Allergy, University Medical Centre Groningen, University of Groningen, P.O. Box 30.001, 9700 RB Groningen, The Netherlands; GRIAC Research Institute, University Medical Centre Groningen, University of Groningen, Groningen, The Netherlands; Department of General Practice, University of Groningen, University Medical Centre Groningen, Groningen, The Netherlands

**Keywords:** Anaphylaxis, Epinephrine auto-injector, Food allergy, General practitioner, Primary care

## Abstract

**Background:**

The knowledge of general practitioner(s) (GPs) regarding food allergy and anaphylaxis and practices in the prescription of epinephrine auto-injector(s) (EAIs) among GPs has previously only been studied using questionnaires and hypothetical cases. Therefore, there are currently no data as to whether or not GPs prescribe EAIs to high risk food-allergic patients presenting to primary care practices. The aim of this study was therefore to describe and evaluate practice in EAI prescription by GPs to food-allergic patients in The Netherlands.

**Methods:**

Patients aged 12–23 years who consulted their GP for allergic symptoms were identified in a primary care database. Patients were classified as probably or unlikely to be food-allergic. A risk factor assessment was done to identify probably food-allergic patients at high risk for anaphylaxis to assess the need for an EAI.

**Results:**

One hundred forty-eight out of 1015 patients consulted their GP for allergic symptoms due to food. Eighty patients were excluded from analysis because of incomplete records. Thirty-four patients were classified as probably food-allergic. Twenty-seven of them were considered high risk patients and candidates for an EAI. Importantly, only 10 of them had actually been prescribed an EAI by their GP.

**Conclusions:**

This study shows that high risk food-allergic patients that visit their GPs are often not prescribed an EAI. Thus, previously identified low rates of EAI ownership may be partly due to GPs not prescribing this medication to patients for whom it would be appropriate to do so. These data suggest that there is a need for improvement of the quality of care for high risk food-allergic patients in primary care.

## Background

General practitioners (GPs) play an important role in diagnosing and treating food-allergic patients. In The Netherlands, the GP is the gatekeeper of the Dutch health-care system controlling access to specialized medical care.

Previous studies have shown that many high risk food-allergic patients do not have an epinephrine auto-injector (EAI) and that GPs are not always knowledgeable about these patients [1–10]. These studies show that there is a lack of allergy knowledge in primary care, especially the recognition and treatment of anaphylaxis were problematic and that national guidelines were often not followed.

Adolescents are the age-group with the highest risk for food allergy fatalities [11]. The fact that they often engage in risk-taking behaviors [12–14] resulting in reduced vigilance about food consumption or reluctance to carry their EAI may contribute to this outcome [13–16].

The knowledge of GPs regarding food allergy and anaphylaxis, and practices in the prescription of EAIs among GPs, has previously only been studied using questionnaires and hypothetical cases. Therefore, there are currently no data as to whether GPs actually do prescribe EAIs to high risk food-allergic patients presenting to primary care practices. The aim of this study was therefore to describe and evaluate practice in EAI prescriptions by GPs to food-allergic patients in The Netherlands.

## Methods

### Study design

A retrospective analysis was performed on data from the electronic database of Registration Network Groningen (RNG). This general practice-based research network was established in 1989 and consists of patient registrations of three group practices based in the Northern part of The Netherlands. The RNG includes a dynamic population with an average annual population of approximately 30,000 patients.

Participating general practitioners use a structured medical record, in which all patient contacts are registered. This includes reason for encounter, medical diagnosis (according to the International Classification of Primary Care (ICPC), applied treatment including prescriptions, using the Anatomical Therapeutic Chemical (ATC) codes, and referrals.

From the RNG database, of the target study population (patients between 12 and 23 years old), a dataset was extracted with information about patients’ consultations for symptoms related to allergy and prescriptions of EAIs by GPs from 2001 to 2012. In this study, patients with allergic symptoms were identified using the ICPC-codes A12 (allergy), T04 (feeding problem of infant/child), and T05 (feeding problem of adult). The EAI prescription was defined as the ATC-group C01CA24 (epinephrine). The database included the following additional information: date of birth, date of entry in the general practice database, GP code, type and number of patient contacts (ICPC codes), prescriptions (ATC codes) and ICPC codes associated with these medications, and (hospital) referrals.

### Study population

The study population consisted of patients aged 12–23 years who consulted their GP for allergic symptoms due to food from 2001 to 2012. Access to the patient’s medical history was a prerequisite. Patients were excluded from analyses if they did not have allergic symptoms to food or if no allergic symptoms were reported in medical record.

### Classification

#### Food allergy

Patients’ medical records were evaluated to identify patients who experienced allergic symptoms solely to food.

Patient age and gender, suspected food(s), allergic symptoms at time of allergic reaction (37 specific symptoms of the mouth, nose, eyes, skin, gastrointestinal tract, respiratory tract, cardiovascular tract), time of onset and duration of allergic symptoms, atopic co-morbidities (asthma, atopic dermatitis, rhinitis), possession of an EAI, and information about other diagnostic tests, and (hospital) referral(s) were determined.

Patients were classified as probably food-allergic when the patient’s medical record indicated that the patient reported allergic symptoms after eating one of the following foods: peanut, tree nuts, milk, egg, wheat, soy, sesame seed, fish, shell fish, and celery. The foods chosen as allergenic food were based on the EU directive on labeling of pre-packaged products (EU Directive 2003/89/EC amending Directive 2000/13/EC) relevant in The Netherlands, i.e. peanut, nuts, milk, egg, wheat, soy, sesame seed, fish, shell fish, and celery. In addition, in patients also reporting reactions to fruits and vegetables, these reactions were documented.

Symptoms of the mouth, nose, eyes, skin, gastrointestinal tract, respiratory tract, or cardiovascular tract were considered allergic symptoms. The presence of only subjective gastrointestinal symptoms without other symptoms was not classified as food allergy.

#### Risk factor assessment and EAI indications

To identify patients who are at high risk for anaphylaxis and to assess the need for an EAI a risk factor assessment based on the guidelines of the European Academy of Allergy and Clinical Immunology (EAACI) was carried out for each patient who experienced allergic symptoms due to food [17].

Risk factors for an anaphylactic reaction were defined as a previously severe anaphylactic reaction to a food requiring emergency treatment or hospitalization as a result, asthma or asthmatic reactions to food, adolescent or young adult age, systemic reaction to traces of the food allergen, and having a peanut or nut allergy [18]. When the first factor was present or when at least two of the other risk factors were present in the context of suspected or proven food allergy, food-allergic patients were considered high risk patients, and candidates for an EAI. Allergy only to fruits or vegetables with these risk factors did not constitute an indication for an EAI. All doubtful cases concerning food allergy and the need for an EAI were discussed with an allergist (AEJD).

#### Referrals

Indications for referral of a patient to a specialist with specific expertise on allergology were defined as patients who experienced allergic symptoms in association with food exposure and who need further testing for the objective diagnosis of food allergy, patients who experienced a severe allergic reaction, and patients being prescribed an EAI [17, 19, 20].

### Statistics

Data entry and analyses were conducted using SPSS version 20.0 (SPSS Inc. Chicago, IL, USA). For the statistical analysis descriptive methods were used.

### Ethical approval

We received ethical permission to access the Registration Network Groningen from the Medical Ethical Review Board, University Medical Center Groningen (UMCG) (METc 2012/366).

## Results

### Classification

#### Food allergy

**Fig. 1:**
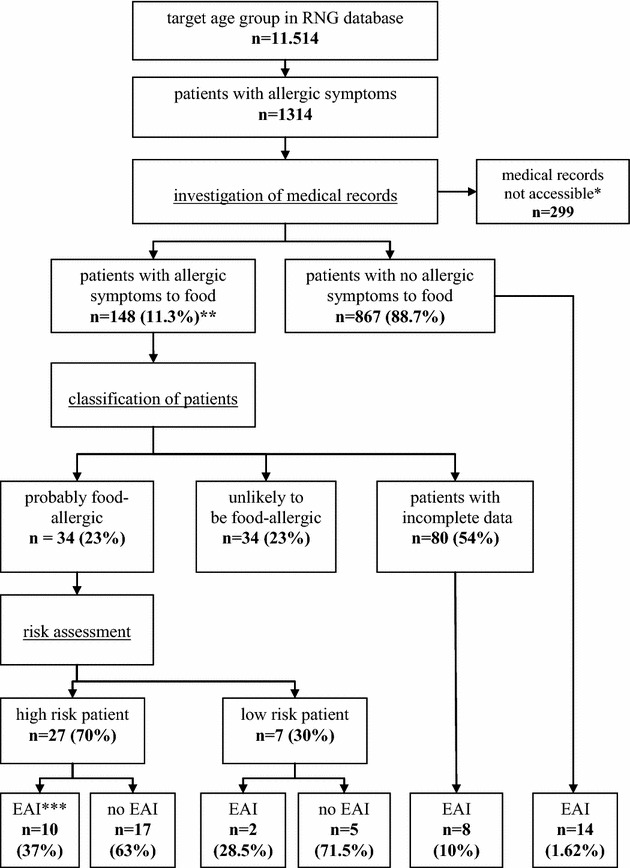
Flow chart of study *Medical records were not accessible because they could not be digitally restored from the archives; **Percentages represent those of the previous step in the chart; ***EAI, epinephrine auto-injector

In total there were 11,514 patients aged 12–23 years identified in the RNG database. Of these 11,514 patients, 1314 patients (11.4 %) consulted their GP for allergic symptoms from 2001 to 2012 (Fig. 1). In total 299 patients’ medical records were not accessible because they could not be digitally restored from the archives. Therefore, a total of 1015 patients were eligible for analysis.

In total 148 patients (14.6 %) out of 1015 patients consulted their GP for allergic symptoms due to food. Of these 148 patients, 80 patients were classified as ‘patients with incomplete data’ because no allergic symptoms were recorded by GP in the patient’s medical records. Of the remaining 68 patients, half (n = 34) were classified as probably food-allergic, and the other half as unlikely to be food-allergic.

Most patients classified as probably food-allergic reported symptoms from tree nuts (44.1 %), milk (32.4 %) and peanuts (23.5 %). Most patients classified as unlikely food-allergic reported symptoms from milk (26.5 %). Descriptive characteristics are shown in Table 1.

**Table 1:** Descriptive characteristics of study population

	Probably food-allergic	Unlikely food-allergic	Patients with incomplete data
Number participants, n (%)	34 (50)	34 (50)	80
Sex adolescent, boys/girls n (%)	17/17 (50/50)	13/21 (38/62)	43/37
Type of food allergies, n (%)			
Peanut	8 (23)	4 (12)	13 (16)
Tree nuts	15 (44)	4 (12)	12 (15)
Cow’s milk	11 (32)	9 (27)	38 (48)
Egg	2 (6)	2 (6)	1 (1)
Wheat	1 (6)	–	0
Soy	–	–	1 (1)
Sesame seed	1 (3)	–	0
Fish	–	2 (6)	1(1)
Shell fish	–	1 (3)	1 (1)
Celery	–	1 (3)	1 (1)
Fruit	–	8 (24)	15 (19)
Vegetables	–	2 (6)	2 (3)
Other	–	7(21)	3 (4)
Tests used to diagnose food allergy, n (%)			
Food-specific IgE levels (RAST)	14 (59)	14 (59)	20 (25)
Open food challenge	9 (27)	7 (21)	9 (11)
Skin prick test	2 (6)	–	1 (1)
Referral to, total n (%)	11 (32)	7 (21)	9 (11)
(Pediatric) allergologist	2 (6)	1 (3)	–
Dermatologist	–	1 (3)	1 (1)
Pediatrician	2 (6)	2 (6)	1 (1)
Internist	4 (12)	3 (9)	1 (1)
Dietician	2 (6)	4 (12)	5 (6)
High risk patient, n (%)	27 (79)	–	4 (5)^b^
Experienced anaphylaxis, n (%)	5 (15)	–	4 (5)
Risk factors, n (%)			
Asthma or asthmatic reactions to food	10 (29)	3 (9)	9 (11)
Adolescent or young adult age	21 (62)	24 (71)	70 (88)
Systemic reaction to traces of the food allergen^a^	6 (18)	1 (3)	–
Having peanut or nut allergy	23(68)	8 (24)	25 (32)
Prescribed an EAI, yes/no n (%)			8 (10)
High risk patient	10/17 (29/71)	–	1 (1)
Low risk patient	2/8 (6/24)	–	7 (9)

EAI epinephrine auto-injector

^a^Not documented in all patient’s medical records

^b^Unknown due to incomplete data, however, 4 patients had had presented to their GP with an anaphylactic reaction according to the information in the medical record. All of them had a peanut or nut allergy, and only 3 of them had been prescribed an EAI

#### Risk factor assessment and EAI indications

Thirty-four patients (3.3 %) out of 1015 patients who consulted their GP for allergic symptoms between 2001 and 2012 were prescribed an EAI. Twenty EAIs were prescribed to patients who consulted their GP for allergic symptoms due to food. The other 14 EAIs were prescribed to five patients who consulted their GP for allergic symptoms due to insect stings, to one patient on recommendation by a pediatrician, and to eight patients of whom the reason of prescription was not clearly recorded in the patient’s medical records.

#### High risk patients and EAIs

Twenty-seven (79.4 %) out of the 34 patients classified as probably food-allergic were considered high risk patients, and therefore candidates for an EAI. Ten (37 %) of them had actually been prescribed an EAI.

Five out of the 27 high risk patients experienced a previous anaphylactic reaction to food. All of these patients were aged 12–17 years, had a peanut and/or tree nut allergy and had asthma. Two (40 %) of them had been prescribed an EAI. One patient’s medical record showed that instructions/demonstrations had been given about when and how to use an EAI.

#### Low risk patients and EAIs

Seven (20.6 %) out of the 34 patients classified as probably food-allergic were considered low risk patients, and therefore not considered candidates for an EAI. Two (28.5 %) of them were prescribed an EAI. One patient had a sesame seed allergy and the other patient a fruit allergy, and both patients reported having asthma.

#### Patients with incomplete data and EAIs

Eighty patients were excluded from analysis in this study because no allergic symptoms were recorded in the patients’ medical records. In the records of four of these patients, it was remarked that they presented to the primary care practice with anaphylactic symptoms after eating food possibly containing peanuts or tree nuts. According to the risk factor assessment used for this study these patients were also considered high risk patients and candidates for an EAI. Importantly, one of them had not been prescribed an EAI. Descriptive characteristics are shown in Table 1.

The other 76 patients of which allergic symptoms were not recorded in their medical record, a risk assessment could not be made nor could the need of an EAI be assessed. In the records of five of these 76 patients, it was remarked that they had been prescribed an EAI. In total eight EAIs were prescribed to patients with incomplete data.

#### Referrals

Twelve patients (32.4 %) (ten high risk and two low risk patients) classified as probably food-allergic were referred to a dietician and/or to one or more specialist(s). Of the other 22 probably food-allergic patients who were not referred, six were prescribed an EAI and one patient experienced anaphylactic symptoms.

Seven patients (20.6 %) classified as unlikely to be food-allergic were referred to a dietician and/or to one or more specialist(s).

None of the patients with incomplete data who experienced anaphylactic symptoms were referred to a specialist.

## Discussion

Although the knowledge of GPs regarding food allergy and anaphylaxis has previously been studied using questionnaires and hypothetical cases, this study is the first to examine actual epinephrine auto-injector prescription practices and the first to make use of data collected by GPs themselves.

Food-allergic patients aged 12–23 years at high risk for anaphylaxis who consult their GPs are often not prescribed an EAI by the GP. Twenty-seven (79.4 %) out of the 34 patients classified as probably food-allergic were considered high risk patients, and therefore candidates for an EAI. Only ten (37 %) of them had actually been prescribed an EAI. In addition, five (18.5 %) out of the 27 high risk patients experienced a previous anaphylactic reaction to food. All of these patients were aged 12–17 years, had a peanut and/or tree nut allergy and had asthma, and only two of them had been prescribed an EAI. This shows that previously identified low rates of EAI ownership may be partly due to GPs not prescribing this medication to patients for whom it would be appropriate to do so [1, 8–10, 21].

The clinical history is a key part of the diagnostic work-up of suspected food allergy [17, 18]. Although the food allergy guidelines of the Dutch College of General Practitioners (NHG) [20] recommends asking the patient about the symptoms and how long these occurred after ingestion, this study shows that many patients’ medical records were lacking this important information. This under-documentation might be due to the patients (or their parents) not clearly remembering or reporting their symptoms. Also, the limited time available to the GP to record the patient’s symptoms during a consultation might play a role. Finally, knowledge and/or practice behavior gaps in GPs might contribute, as is suggested by other studies [4–6, 9, 22].

Primary care guidelines in The Netherlands, the NHG guidelines [20], recommend that an EAI should only be prescribed after a previous case of anaphylaxis. Significantly, risk factors for a life-threatening food induced anaphylactic reaction are mentioned, but are not put forward as an reason to prescribe an EAI in the absence of a previous anaphylactic reaction. Although it may therefore be argued that GPs are simply following their own guidelines, this does not seem to explain the lack of EAI prescription in 3 out of 5 high risk patients who had experienced prior anaphylaxis as well as 3 out of 4 patients presenting with anaphylaxis to their GP. The latter situation is in agreement with previous studies of anaphylaxis management in emergency rooms, where patients presenting with anaphylaxis are not always prescribed an EAI or referred to appropriate specialist care allergy [17, 18].

The need for improved EAI training has been a recurring theme in the literature on anaphylaxis [8, 11, 13, 23, 24]. Anaphylaxis usually occurs in the community, therefore, all food-allergic patients and their parents (and other caregivers) should be provided with educational resources and training should cover avoidance strategies, recognition of symptoms, and when and how to administer an EAI. It was mentioned in one patient’s medical record that EAI instructions/demonstrations had been given. The NHG guidelines recommend giving clear instructions about the use of an EAI. It may thus be possible that GPs did give clear instructions about when and how to use an EAI, but that this was not documented. The general practices did not have demonstration material and trainer EAIs on hand. We found that GPs feel that giving instructions about how to use an EAI is the responsibility of the pharmacist. Further research is needed to investigate the quality of the EAI training currently offered by GPs and pharmacists.

In this study, the majority of patients classified as probably food-allergic were not referred for further testing for an objective diagnosis of food allergy. It should be noted that there is good availability of specialist allergy care for pediatric patients in the area where this study was conducted. Infrequent or no referrals of patients to a specialist with specific expertise on allergology after an allergic reaction is in agreement with the findings in previous studies [4, 5]. One might speculate that GPs do not recognize allergic reactions or underestimate their severity, and therefore do not refer patients for further diagnosis. Implementing clear referral criteria could be beneficial to assist GPs to refer patients to a specialist with specific expertise on allergology when this is needed. In the Northern part of The Netherlands our centre and other hospitals can be reached easily by car or public transportation. All patients in The Netherlands have health insurance and all costs are covered. In our centre we have a waiting list for food challenges. However, all patients are seen within 2 weeks at the out-patient clinic. When necessary they are prescribed an EAI.

In contrast to these results, inappropriate prescription or referral of patients who could conceivably be managed without an EAI in primary care seems to be numerically less of a problem. However, given the impact that overestimation of risk may have on patient well being, over-treatment should be a target for improvement of quality of care as well as under-treatment.

Strength of this study is that, to our knowledge, this is the first study to assess the epinephrine auto-injector prescription practices of general practitioners to food-allergic patients at high risk for anaphylaxis using data recorded by GPs themselves.

A limitation in this study is the number of patients excluded from analysis due to inaccessible or incomplete patient’s medical records. The incomplete data might be due to under-reporting of patients (or their parents), under-documentation of clinical information by GPs or lack of knowledge and/or practice behavior gaps experienced by GPs. In this regard, it may be expected there would be more food-allergic patients at high risk for anaphylaxis not being prescribed an EAI. We did not find patients who had been prescribed an EAI who were not high risk patients. Ultimately, some of these patients would probably show no reactions when challenged with the food in question. However, such patients would still require an EAI until challenge tests could be done, and overestimation of the need for EAIs would thus only be apparent after such tests had been completed. Our findings may thus eventually be an overestimation of the problem of inadequate EAI prescription to high-risk food-allergic patients to some degree. More urgent, however, is the obvious extent to which high risk food allergic patients are not prescribed an EAI by their GPs.

In summary, although previous studies have shown that some high risk food-allergic patients do not seek medical care, this study shows with data recorded by GPs themselves that those that do visit their GPs are often not prescribed an EAI, even to those with a previous severe anaphylactic reaction. This shows that previously identified low rates of EAI ownership may be partly due to GPs not prescribing this medication to patients for whom it would be appropriate to do so. These data suggest that there is a need for improvement of the quality of care for high risk food-allergic patients in primary care.

## Conclusions

Food-allergic patients at high risk for anaphylaxis who visit their GP are often not prescribed an epinephrine auto-injector. There is a need for improvement of the quality of care for high risk food-allergic patients in primary care.

## Abbreviations

ATC: Anatomical Therapeutical Chemical
EAACI: European Academy of Allergy and Clinical Immunology
EAI(s): epinephrine auto-injector(s)
GP(s): general practitioner(s)
ICPC: International Classification of Primary Care
NHG: Dutch College of General Practitioners
RNG: Registration Network Groningen
